# Targeting of the Interleukin-13 Receptor (IL-13R)α2 Expressing Prostate Cancer by a Novel Hybrid Lytic Peptide

**DOI:** 10.3390/biom13020356

**Published:** 2023-02-12

**Authors:** Riaz Jannoo, Zhidao Xia, Paula E. Row, Venkateswarlu Kanamarlapudi

**Affiliations:** 1 UCL ECMC GCLP Facility, UCL Cancer Institute, University College London, London WC1E 6DD, UK; 2 Institute of Life Science, School of Medicine, Swansea University, Singleton Park, Swansea SA2 8PP, UK

**Keywords:** prostate cancer, IL-13Rα2, hybrid lytic peptide, Pep-1, Phor21, therapeutic peptide, 3D spheroids, epigenetics

## Abstract

The IL-13Rα2 cell surface receptor is highly expressed in tumours such as prostate cancer. In this report, we evaluated the hypothesis that prostate cancer cells with enhanced IL-13Rα2 expression are a suitable target for the hybrid lytic peptide (Pep-1-Phor21) peptide, which is generated by fusing the IL-13Rα2 specific ligand (Pep-1) and a cell membrane disrupting lytic peptide (Phor21). The expression of IL-13Rα2 mRNA and protein in prostate cancer tissues and cell lines was assessed via real-time PCR (RT-PCR) and immunoblotting. The effect of Pep-1-Phor21 on the viability of prostate cancer cells grown in monolayers (2D) and microtissue spheroids (3D) was assessed via CellTox green cytotoxic assay. IL-13Rα2 expression and Pep-1-Phor21-mediated killing were also determined in the cells treated with epigenetic regulators (Trichostatin A (TSA) and 5-aza-2 deoxycytidine (5-Aza-dC)). The hybrid lytic peptide cytotoxic activity correlated with the expression of IL-13Rα2 in prostate cancer cell lines cultured as monolayers (2D) or 3D spheroids. In addition, TSA or 5-Aza-dC treatment of prostate cancer cells, particularly those with low expression of IL-13Rα2, enhanced the cells’ sensitivity to the lytic peptide by increasing IL-13Rα2 expression. These results demonstrate that the Pep-1-Phor21 hybrid lytic peptide has potent and selective anticancer properties against IL-13Rα2-expressing prostate cancer cells.

## 1. Introduction

Prostate cancer is the second most diagnosed cancer type and the fifth leading cause of cancer death in men worldwide [1]. Like most solid tumours, prostate cancer is a highly lethal tumour that can metastasise to distant organs such as the bone, liver, lungs and brain if not detected early [1]. Patients diagnosed with prostate cancer at its early stages have a 5-year survival rate of 100% [1]. However, the 5-year survival rate drops significantly to 31% in patients diagnosed with advanced stages of the disease [1]. Current treatments (such as chemotherapy) cannot cure but only prolong patients’ lives. Chemotherapeutic treatment is usually given when cancer becomes metastatic [2]. Although chemotherapeutic drugs are effective, they destroy both rapidly dividing cancerous and non-cancerous cells, and hence their use can cause serious side effects by destroying healthy tissue and organs. Moreover, these drugs are unable to target dormant cancer cells and slow-growing tumours [3]. The effectiveness of these chemotherapeutic drugs is further reduced once the cancer cells overexpress drug efflux pumps such as P-glycoprotein, developing resistance to the treatment [4,5]. Although hormonal therapy has been shown to reduce tumour size in most men with advanced prostate cancer, with few side effects, its use can cause the disease to re-emerge and differentiate into a more aggressive form, making the treatment ineffective [4,6]. These studies clearly illustrate the need for the development of novel effective treatments for metastatic prostate cancer, with fewer or no side effects.

The cytokine Interleukin (IL)-13 plays an important role in allergy and atopic diseases [7]. It has two receptors, a low-affinity IL-13Rα1 and a high-affinity IL-13Rα2 [8,9,10]. IL-13Rα1 heterodimerizes with the IL-4 receptor (IL-4R)α bind to and mediate IL-13 cellular functions. The heterodimeric receptor IL-13Rα1/IL-4Rα is widely expressed in normal tissues, whereas IL-13Rα2 has a limited normal tissue distribution [11]. IL-13Rα2 is also known as cancer/testis antigen (CT)19 since it is one of the testis-specific antigens that is overexpressed in a range of solid cancers, and, therefore, it can serve as a potential target for cancer therapy [12,13,14,15]. IL-13Rα2 was initially thought to be a decoy receptor since it was shown not to mediate IL-13-induced cellular responses or to activate any downstream signalling on its own [16]. However, further studies revealed IL-13-dependent and IL-13-independent functions for IL-13Rα2, suggesting that the receptor may be more than a decoy receptor [17,18,19]. More recently, IL-13Rα2 has been shown to mediate signal transduction through the Src/PI3K/Akt/mTOR and AP1 signalling pathways as well as the MAP kinase and STAT3 pathways when it binds to EGFRvIII [20,21,22,23,24]. IL-13Rα2 promotes glioblastoma multiforme (GBM) invasiveness and proliferation, as well as acting as a negative prognostic marker in lung cancer and luminal invasive subtype breast cancer [20,23,25]. Therefore, IL-13Rα2 has gathered a lot of interest as a possible drug target for treating cancer [26]. A cytotoxic drug composed of IL-13 and a modified bacterial toxin, *Pseudomonas* exotoxin 38 (IL-13-PE38), has been developed for GBM therapy, as up to 80% of cases of GBMs show overexpression of IL-13Rα2 [27]. However, phase III clinical trials on IL-13-PE38 have shown that this drug can be toxic at high doses since it can also target IL-4Rα/IL-13Rα1 heterodimers [28,29,30].

Several tumour suppressor and other cancer-related genes have been identified to be inactivated via the methylation of CpG islands in their promoter region [31,32]. Histone deacetylation has also been found to be associated with transcriptional silencing through chromatin condensation [33,34,35,36]. There is increasing evidence to suggest that epigenetic alterations, such as histone acetylation and promoter DNA methylation, play an important role in the regulation of gene expression of IL-13Rα2 [37,38]. Trichostatin A (TSA), a histone deacetylase inhibitor, and 5-aza-2 deoxycytidine (5-Aza-dC), a DNA methyltransferase inhibitor, have demonstrated their potential in anticancer treatments [39]. The histone deacetylase inhibitor and DNA methyltransferase inhibitors have been shown to upregulate IL-13Rα2 expression in pancreatic cancer [38].

We have developed a novel hybrid lytic peptide (Pep-1-Phor21) to specifically target cancer cells overexpressing IL-13Rα2. The Pep-1-Phor21 hybrid peptide is composed of an IL-13Rα2 binding ligand, Pep-1 and a cell-membrane-disrupting lytic peptide called Phor21 [40,41]. Pep-1 is a seven amino acid [42] residue peptide (GEMGWVR), which was first isolated by screening for IL-13Rα2 ligands using a C7C phage display library [40]. It not only specifically binds IL-13Rα2 with high affinity but was also shown to bind at a site different from that of IL-13 on IL-13Rα2. As a result, the in vitro binding of Pep-1 to IL-13Rα2 is not inhibited by IL-13. Therefore, it is assumed that IL-13 and Pep-1 will not inhibit each other’s ability to bind to IL-13Rα2 under physiological conditions. Similarly, Pep-1 neither binds to the IL-13Rα1/IL-4Rα complex nor inhibits IL-13 binding to IL-13Rα1/IL-4Rα [40]. Therefore, unlike IL-13-PE38, Pep-1-conjugated drugs have the potential to specifically target IL-13Rα2-overexpressing tumours [40]. Moreover, Pep-1-conjugated nanoparticles and the α-particle emitter Actinium-225 have recently been shown to target IL-13Rα2-overexpressing GBM, indicating the peptide’s potential in drug-targeting GBM [43,44]. Phor21 is a well-characterised lytic peptide, which consists of three repeats of Heptad (a seven-amino-acid-long peptide composed of mainly positively charged lysine residues (KFAKFAK)) [45]. It is a linear amphipathic α-helical cationic peptide that binds negatively charged plasma membrane, causing necrotic cell death through lysing the membrane [46]. The lytic peptide that kills cells via apoptosis internalises, whereas the lytic peptide that elicits necrosis does not [47]. Furthermore, the lytic peptide, which causes necrosis, can bypass multidrug resistance since it acts on the cell membrane [46]. Although Phor21 lytic peptide is more effective at killing cancer cells (with a high negative membrane charge) than non-tumour cells, the peptide efficacy can be improved by conjugating it to ligands of the cell surface receptors that are known to be overexpressed in cancer cells [46,48]. Previous studies using Phor21 covalently conjugated to a peptide derived from the β-chain of chorionic gonadotropin (βCG) (Phor21-βCG[ala]) showed a significant decrease in the size of tumours that overexpress the luteinising hormone/CG receptor (LHCGR) [41,49]. A previous study on pharmacokinetic analysis of Phor21-βCG[ala] revealed that it has an in vivo half-life of 5 h [50]. With a short systemic half-life, it is very unlikely that this peptide elicits an immune response and/or causes liver toxicity. However, no such studies have been carried out so far to analyse the anticancer effects of Pep-1 conjugated to a lytic peptide. A hybrid peptide, consisting of an IL-13Rα2-binding peptide (A2b11) and a lytic peptide, was demonstrated to have anti-tumour activity against glioblastomas in mice [51]. By specifically inhibiting the IL-13Rα2 signalling pathway, a 12aa peptide has been shown to reduce GBM and colorectal cancer cell migration and invasiveness [52].

In this paper, we have studied the effect of Pep-1-Phor21 on the viability of prostate cancer cells grown in a monolayer (2D) and as 3D spheroids, which can resemble living tumours. For this purpose, we first focused on analysing the expression of IL-13Rα2 in prostate cell lines, testing the efficacy of the hybrid lytic peptide (Pep-1-Phor21) in killing prostate cancer cells in vitro and the relationship between the prostate cell lines’ sensitivity to Pep-1-Phor21 and their IL-13Rα2 expression. We next determined whether the sensitivity of the cancer cells to the hybrid lytic peptide could be altered by upregulating IL-13Rα2 expression by treatment with epigenetic inhibitors. Finally, we analysed IL-13Rα2 expression and sensitivity to Pep-1-Phor21 of 3D-cultured prostate cancer cells treated without or with epigenetic inhibitors.

## 2. Materials and Methods

### 2.1. Antibodies and Other Reagents

Anti-IL-13Rα2 (sc-134363) mouse monoclonal was obtained from Santa Cruz Biotechnology (Dallas, TX, USA). Horseradish peroxidase (HRP)-conjugated secondary antibodies and enhanced chemiluminescence (ECL) advanced reagent were from Cytiva (Little Chalfont, UK). Pep-1 (CGEMGWVRC), Phor21 (KFAKFAKKFAKFAKKFAKFAK), Pep-1-Phor21 (CGEMGWVRCKFAKFAKKFAKFAKKFAKFAK) and Phor21-βCG(ala) (KFAKFAKKFAKFAKKFAKFAKSYAVALSAQAALARR) were synthesised by Thermo Fisher Scientific (Loughborough, UK). AlamarBlue was obtained from Thermo Fisher Scientific (Loughborough, UK). CellTox Green and ApoTox-Glo Triplex assay kits were purchased from Promega (Southampton, UK). The pharmacological inhibitors TSA, 5-AZA and an anti-α tubulin mouse monoclonal were from Merck (Gillingham, UK). JetPRIME transfection reagent was obtained from Polyplus-transfection (Illkirch, France). All other reagents, unless otherwise specified, were obtained from Merck (Gillingham, UK).

### 2.2. Cell Culture

Human prostate epithelial non-cancer (PNT2) and cancer cell lines (LNCaP, DU145 and PC-3) obtained from American Type Culture Collection (ATCC) (Rockville, MD, USA) were cultured in *RPMI*-*1640* supplemented with 10% foetal bovine serum (FBS), 2 mM L-glutamine, 100 µg/ mL penicillin and 100 µg/ mL streptomycin (complete medium) at 37 °C/5% CO_2_ in a humidified incubator.

### 2.3. Cell Transfection

Cells were transfected with expression plasmid FLAG-IL-13Rα2 [53], Myc-LHCGR [54] or an empty control plasmid (pcDNA3) using JetPRIME transfection reagent according to the manufacturer’s instructions. Briefly, 200 µL of JetPRIME buffer containing 2 µg of plasmid DNA and 4 μL JetPRIME transfection reagent were added to cells grown to 60–80% confluency in a 6 cm plate. The cells were used for experimentation 2 days after transfection.

### 2.4. Real-Time Polymerase Chain Reaction (RT-PCR)

Total RNA was isolated from cultured cells by using the RNeasy mini kit (Qiagen [Manchester, UK]) according to the manufacturer’s protocol. One µg of total RNA was reverse transcribed to complementary DNA (cDNA) in 20 µL reaction volume using the High-Capacity cDNA Reverse Transcription Kit (Applied Biosystems [Warrington, UK]) and by following the supplier instructions. The RT-PCR was carried out in a final volume of 10 µL containing 0.25 µL cDNA, 250 nM of both the forward and reverse primers and SensiFAST SYBR & Fluorescein Master Mix (Bioline [London, UK]), using the CFX 96 real-time detection system (Bio-Rad [Watford, UK]). The primers used for RT-PCR were 5′-TAACCTGGTCAGAAGTGTGCC-3′ (sense) and 5′-GGAGGGTTAACTTTTATACTCGGTGT-3′ (antisense) for IL-13Rα2 or 5′-CAGCCATGTACGTTGCTATCCAGG-3′ and 5′-AGGTCCAGACGCAGGATGGCATG-3′ (antisense) for β-actin. The assay was conducted in triplicate for each sample and the relative expression of IL-13Rα2 mRNA was calculated using the 2^−ΔΔCt^ method and β-actin mRNA as the internal control [55]. The IL-13Rα2 gene expression in prostate cancer tissue was analysed via RT-PCR using Origene TissueScan prostate cancer cDNA array (20 cancer tissue samples and 4 normal (non-cancer) tissue samples) [56].

### 2.5. Protein Extraction

The cells were thrice washed with cold phosphate-buffered saline (PBS) and lysed using radioimmunoprecipitation assay (RIPA) lysis buffer (10 mM Tris-HCl, pH 7.5, 10 mM EDTA, 1% NP-40, 0.1% SDS, 150 mM NaCl and 0.5% sodium deoxycholate) containing 1% mammalian proteinase inhibitor cocktail. The lysate was subsequently passed through a 25-gauge needle ≥ 10 times to shear chromosomal DNA and was centrifuged at 22,000× *g* for 10 min at 4 °C to pellet cell debris and unlysed cells. The supernatant was mixed with 25% of 5× SDS-polyacrylamide gel electrophoresis (PAGE) sample loading buffer (5% SDS, 125 mM Tris-HCl, pH 6.6, 50% glycerol, 0.025% bromophenol blue, 5% 2-Mercaptoethanol), boiled at 100 °C for 5 min and stored at −20 °C until further use.

### 2.6. Immunoblotting

Briefly, proteins fractionated via SDS-PAGE gel electrophoresis were transferred onto a polyvinylidene fluoride (PVDF) membrane [57]. Membranes were blocked with TBST (Tris-buffered saline (TBS) with 0.1% tween 20) containing 5% milk powder (blocking buffer) for 1 h at room temperature and then incubated with anti-IL-13Rα2 mouse monoclonal (diluted 1:500 in blocking buffer) overnight at 4 °C. The membranes were then washed with TBST and incubated with the HRP-conjugated anti-mouse secondary antibody (diluted 1:2500 in blocking buffer) for 1 h at room temperature. Membranes were then developed using ECL select substrate and bands on the membrane were visualised using a ChemiDoc^TM^ XRS system (Bio-Rad) [58]. Blots were stripped of antibodies by incubating them in Western blot stripping buffer at RT for 15 min. The blots were then washed, blocked and re-probed using anti-α tubulin mouse monoclonal antibody (diluted 1:10,000) and HRP-conjugated anti-mouse secondary antibody (diluted 1:5000 in blocking buffer) [59].

### 2.7. Enzyme-Linked Immunosorbent Assay (ELISA)

Cell surface expression of IL-13Rα2 was assessed via ELISA using non-permeabilised cells as described [54]. Cells grown to 60–80% confluency in poly-L-lysine-coated wells (0.1 mg/mL) of a 48-well plate were fixed with 4% (*w*/*v*) paraformaldehyde for 5 min and blocked for 45 min by incubating them with blocking buffer (1% bovine serum albumin (BSA) made in TBS (1% BSA/TBS)). Cells were then incubated with anti-IL-13Rα2 mouse monoclonal antibody (diluted 1:800 in 1% BSA/TBS) or isotype control mouse IgG for 2 h. Cells were washed 3 times with TBS and then incubated with HRP-conjugated anti-mouse IgG (diluted 1:5000 in 1% BSA/TBS) for 1 h. Cells were washed 3 times and developed by incubating with 1-step Ultra TMB-ELISA substrate (Bio-Rad) for 15 min, and the reaction was stopped by adding an equal volume of 2 M H_2_SO_4_. The absorbance of the reaction mixture was read at 450 nm using a microplate reader [59]. After deducting the background absorbance (due to incubation with isotype control mouse IgG), the absorbance was normalised by the total number of cells (assessed via crystal violet staining) in each well.

### 2.8. Crystal Violet Staining

Briefly, cells were washed with PBS and stained with 0.2% crystal violet solution, which was made using ethanol. After washing with PBS, the dye was extracted from the stained cells using 1% SDS and absorbance at 570 nm was measured.

### 2.9. Cell Viability Assay

Cell viability was assessed using the alamarBlue assay. Cells in the quantity of 40,000 were plated in each well of a 96-well µclear half area black plate (Greiner Bio-one) and incubated at 37 °C/5% CO_2_ in a humidified incubator. After 24 h, the medium was replaced with the complete medium containing 10% (*v*/*v*) alamarBlue and 0–120 µM of Phor21 or Pep-1-Phor21. The fluorescence of the medium was read 30 min after incubation (considered as zero) and every 3 h afterwards by using a microplate reader (POLAR star Omega) with 570 nm (excitation) and 630 nm (emission) settings.

### 2.10. Cytotoxicity Assay

Cell cytotoxicity was assessed using the CellTox™ Green Cytotoxicity Assay (Promega). Cells in the quantity of 40,000 were plated in each well of a 96-well µclear half area black plate (Greiner Bio-one) and incubated at 37 °C/5% CO_2_ in a humidified incubator. After 24 h, the medium was replaced with the complete medium containing 0.1% (*v*/*v*) CellTox Green Dye and a set concentration of Phor21 or conjugated Phor21 at 37 °C/5% CO_2._ The fluorescence was assessed by using 490 nm (excitation) and 525 nm (emission) filter settings.

### 2.11. ApoTox-Glo^TM^ Triplex Assay

Cell viability, cytotoxicity and apoptosis of PC-3 cells were determined simultaneously using the ApoTox-Glo ^TM^ Triplex assay kit (Promega) according to the manufacturer’s protocol. The cells (40,000 per well) were plated into a 96-well µclear half area black plate and incubated for 24 h at 37 °C/5% CO_2_ in a humidified incubator. The medium in wells was replaced with the complete medium containing the test compound and the cells were incubated for 6 h at 37 °C/5% CO_2_, and then cell viability and cytotoxicity were determined by adding 10 µL of glycylphenylalanyl-aminofluorocoumarin (GF-AFC) substrate or bis-alanylalanyl-phenylalanyl-rhodamine 110 (bis-AAF-R110) substrate, respectively, to each well and incubating the plate for 1 h at 37 °C/5% CO_2_. Cell viability was measured by using 400 nm for excitation and 505 nm for emission in a microplate reader (POLAR star Omega). Cytotoxicity was measured by using 485 nm for excitation and 520 nm for emission. Apoptosis was determined by adding 50 µL of Caspase-Glo 3/7 reagent to each well, incubating for 30 min at room temperature and measuring luminescence using a microplate reader (POLAR star Omega).

### 2.12. TSA and 5-aza-dC Treatment

Cells grown in complete medium to 80% confluency in a 6 cm plate (for immunoblotting and RT-PCR) or in a 96-well µclear half area black plate (for cell viability or cytotoxicity assays) were treated with 0–100 µM TSA or 5-aza-dC. After 24 h of treatment, Phor21 or Pep-1-conjugated Phor21 (Pep-1-Phor21) were added to the cells and the cells were incubated for an additional 3 h at 37 °C/5% CO_2_ [38,60]. They were then used for both RT-PCR and immunoblotting assays or plated for cytotoxicity assays.

### 2.13. Three-Dimensional Culturing

PNT2, LNCaP, DU145 and PC-3 cells grown in monolayers were used for generating cell spheroids, which mimic three-dimensional (3D) culture. Cells were detached with 1× Trypsin/EDTA. The volume of 20 µL (1 × 10^5^ cells/mL) of the cells was seeded into each well of a Terasaki plate (Greiner Bio-one), and the plates were incubated upside down in a humidified incubator to grow the cells as spheroids. After 1 day of incubation, the spheroids along with the medium pooled from 3 wells of the Terasaki plate were transferred into a single well of U-bottom surface repellent 96-well plates (CELLSTAR Greiner bio-one), incubated in a humidified incubator for 1 day and used for cytotoxicity assays. Cell spheroid formation was viewed using an inverted light microscope.

The viability of cells in spheroids was also assessed using LIVE/DEAD staining (Invitrogen). For this, single-cell suspensions (1 × 10^5^/mL) were stained via incubation with fluorescent vital membrane dye Vybrant DiO (Dio, 1:200 dilution) for 10 min in a humidified incubator. The labelled cells were washed with RPMI-1640 and the cell pellets were resuspended in the fresh complete medium to obtain the same cell density. The fluorescently labelled cells were seeded into wells of a Terasaki plate and incubated as described above to generate spheroids. Matrigel (reconstituted basement membrane; BD Biosciences) was thawed on ice overnight and mixed with an equal volume of ice-cold complete medium, and 30 µL of it was pipetted into each well of a 96-well black plate with clear flat bottoms (Greiner Bio-one) and incubated in a humidified incubator for 24 h to allow the Matrigel to solidify. The DiO-stained spheroids were grown in the wells of the Terasaki plate for 24 h, transferred into the Matrigel and incubated for 3 h in the presence or absence of the test compound. Then, the samples were incubated in phenol red-free RPMI-1640 containing 2 µM ethidium homodimer-I (EthD-1) at room temperature for 40 min. The wells containing spheroids in Matrigel were washed three times with phenol red-free RPMI-1640 and immediately imaged in the same medium using a confocal microscope (LSM 710, Carl Zeiss Ltd. [Cambridge, UK]).

### 2.14. Statistical Analysis

Data were analysed using the GraphPad Prism program. All data are presented as means ± standard error of the mean (SEM) of three independent experiments. Statistical tests between controls and test values were performed using a two-tailed unpaired Student’s *t*-test. Statistical tests between groups were performed using Bonferroni’s post-test after one-way or two-way analysis of variance (ANOVA), where *p* > 0.05 was considered as statistically not significant (ns) and *p* ≤ 0.05, *p* ≤ 0.01 and *p* ≤ 0.001 were considered to be statistically significant [59].

## 3. Results

### 3.1. Expression of IL-13Rα2 in Prostate Cancer Tissues and Cell Lines

**Figure 1:**
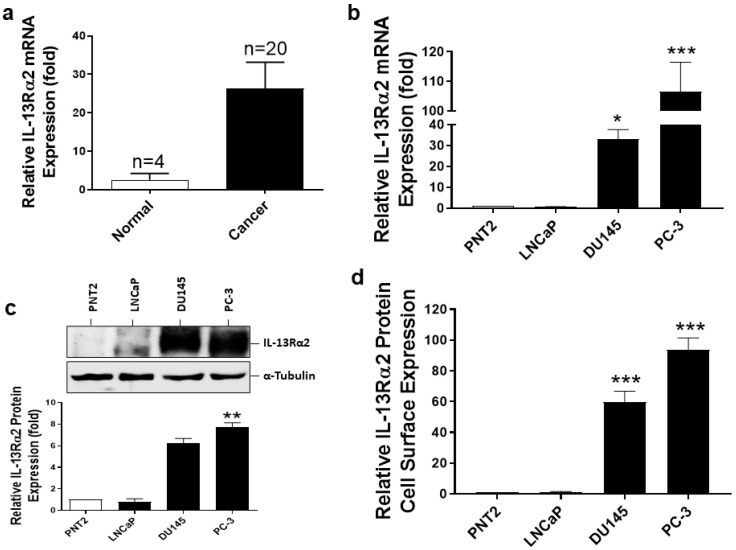
Expression of IL-13Rα2 in prostate tissues and cell lines. (**a**) Relative-fold change in expression of IL-13Rα2 mRNA in normal and cancerous tissues. (**b**) Real-time PCR (RT-PCR) analysis of IL-13Rα2 mRNA expression in non-cancer and cancer cell lines. (**c**) Western blot analysis of IL-13Rα2 protein expression in PNT2, LNCaP, DU145 and PC-3 cells. Quantification of IL-13Rα2 protein expression was performed via densitometric analysis of the bands and normalising to housekeeping protein (α-Tubulin) expression. (**d**) Cell surface expression of IL-13Rα2 protein was assessed by subjecting non-permeabilised cells to ELISA. All Western blots and RT-PCR values are normalised to housekeeping controls. The data are mean ± SEM values of three independent experiments (* *p* < 0.05, ** *p* < 0.01 and *** *p* < 0.001 compared with non-cancer cell line control).

To determine whether the expression of the IL-13Rα2 gene is altered in prostate cancer, we assessed IL-13Rα2 expression at the mRNA level in human prostate cancer tissues using the TissueScan prostate cancer cDNA array via RT-PCR. As shown in Figure 1a, the expression of the IL-13Rα2 gene was significantly higher in prostate cancer tissues than in normal prostate tissues. The expression of IL-13Rα2 mRNA and protein was then analysed in prostate non-cancer (PNT2) and cancer (androgen-dependent (LNCaP) and androgen-independent with a high metastatic potential (DU145 and PC-3)) cell lines via RT-PCR (Figure 1b) and immunoblotting (Figure 1c), respectively. IL-13Rα2 mRNA expression was low but detectable in PNT2 cells and therefore IL-13Rα2 mRNA expression in PNT2 cells was used to compare with that in the prostate cancer cell lines to analyse relative expression. When compared to IL-13Rα2 mRNA expression in PNT2 cells, metastatic DU145 and PC-3 cells, but not non-metastatic LNCaP cells (0.53 ± 0.15-fold), showed high levels of IL-13Rα2 mRNA (32.1 ± 3.1-fold (*p* ≤ 0.05) and 111.5 ± 6.9-fold (*p* ≤ 0.001)) overexpression in DU145 and PC-3 cells, respectively (Figure 1b). Consistent with this, only DU145 and PC-3 cells expressed high levels of the IL-13Rα2 protein when compared to that in PNT2 cells (31.0 ± 2.6-fold (*p* ≤ 0.001) and 29.1 ± 5.0-fold (*p* ≤ 0.001), respectively) (Figure 1c). Since the IL-13Rα2 protein functions mainly at the cell surface, we have also assessed its cell surface expression in the prostate non-cancer and cancer cell lines via cell surface ELISA (Figure 1d). IL-13Rα2 protein cell surface expression is relatively high in metastatic prostate cancer cell lines DU145 and PC-3 (59.6 ± 7.2-fold (*p* ≤ 0.001) and 93.8 ± 7.7-fold (*p* ≤ 0.001) overexpression in DU145 and PC-3, respectively, compared to PNT2). Together, these results suggest that IL-13Rα2 expression is high in prostate cancer tissues and metastatic cell lines.

### 3.2. The Specificity of Pep-1-Phor21 in Targeting IL-13Rα2-Expressing Cells

**Figure 2:**
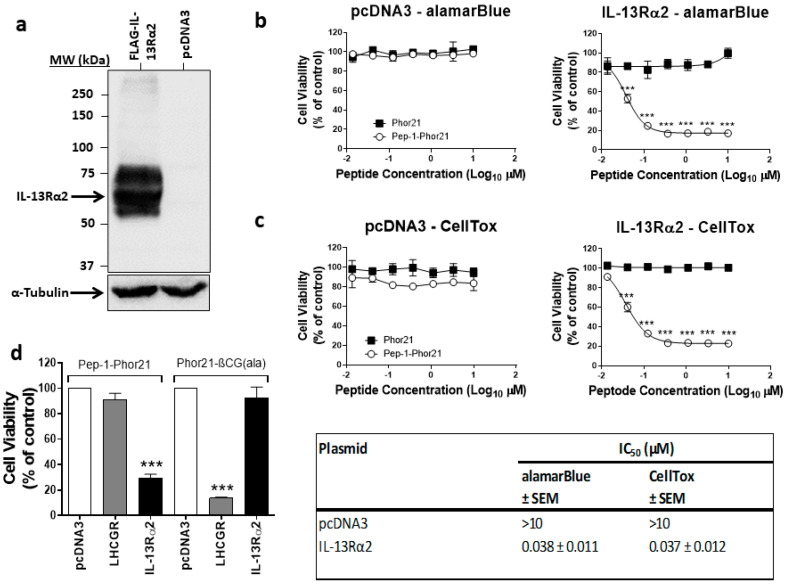
Pep-1-Phor21 specificity in targeting IL-13Rα2-expressing cells. (**a**) Western blot analysis of IL-13Rα2 protein expression in HEK293 cells. The lysates of HEK293 cells transfected with IL-13Rα2 plasmid or an empty plasmid (pcDNA3) were separated via SDS-PAGE, transferred onto the PVDF membrane and probed using an anti-IL-13Rα2 antibody. The lysates were also probed with an anti-α-Tubulin antibody to ensure equal loading. HEK293 cells transfected with pcDNA3 or IL-13Rα2 were incubated with 0 - 10 µM (a 3-fold serial dilution starting with 10 µM (10 µM, 3.3 µM, 1.1 µM, 0.37 µM, 0.12 µM, 0.04 µM, 0.01 µM) was used) of Pep-1-Phor21 (○) or Phor21(■) for 3 h and their cytotoxicity was measured using alamarBlue (**b**) and CellTox (**c**) assays. (**d**) The specificity of the cytotoxicity effect of Pep-1-Phor21 on HEK293 cells expressing IL-13Rα2. HEK293 cells expressing nothing or IL-13Rα2 or LHCGR were incubated with 0.5 µM Pep-1-Phor21 or Phor21-βCG (ala) for 3 h and their cytotoxicity was measured via CellTox assay. The data represent means ± SEM (error bars represent SEM) of three independent experiments (*** *p* < 0.001).

To assess Pep-1-Phor21 specificity in targeting and killing IL-13Rα2-expressing cells, HEK293 cells (IL-13Rα2 -ve) transfected with an empty control plasmid or IL-13Rα2 expression plasmid were used. The lysates of HEK293 cells transfected with these constructs were immunoblotted using an anti-IL-13Rα2 antibody to assess the expression of IL-13Rα2 (Figure 2a). IL-13Rα2 was expressed as an approximately 50 kDa protein in cells transfected with IL-13Rα2 plasmid but not in cells transfected with empty plasmid. HEK293 cells transfected with either empty vector or IL-13Rα2 plasmid were then treated with 0–10 µM lytic peptide Phor21 or Phor21 conjugated to the ligand (Pep-1-Phor21) for 3 h and toxicity was assessed using both cell viability (alamarBlue; Figure 2b) and cytotoxicity (CellTox green; Figure 2c) assays. Pep-1-Phor21 showed dose-dependent cytotoxicity and loss of cell viability only in cells expressing IL-13Rα2, with a 50% inhibitory concentration (IC_50_) of 0.037 µM determined by both methods. The lytic peptide (Phor21) alone did not cause any cytotoxicity or reduced cell viability of HEK293 cells expressing IL-13Rα2, whereas Pep-1-Phor21 had no effect on HEK293 cells transfected with an empty vector. To confirm that Pep-1-Phor21 only targets IL-13Rα2, HEK293 cells expressing IL-13Rα2 or LHCGR were treated with Pep-1-Phor21 or Phor21-βCG(ala), which is a ligand for LHCGR, and assessed for cell viability (Figure 2d). Pep-1-Phor21 did not affect the cell viability of cells expressing LHCGR, whereas Phor21-βCG(ala)-treated LHCGR-expressing cells showed a significant decrease in cell viability (92.87 ± 8.08% (*p* > 0.05) and 13.80 ± 0.62% (*p* < 0.001), respectively). IL-13Rα2-expressing cells showed a significant decrease in cell viability when treated with Pep-1-Phor21, whereas Phor21-βCG (ala) had no effect on the cell viability of IL-13Rα2-expressing cells (29.70 ± 2.76% (*p* < 0.0001) and 91.30 ± 4.85% (*p* > 0.05), respectively). These results demonstrate that Pep-1-Phor21, but not unconjugated lytic peptide or lytic peptide conjugated to an irrelevant ligand, specifically targets IL-13Rα2-expressing cells.

### 3.3. The Cytotoxic Effect of Pep-1-Phor21 Peptide on Prostate Cancer Cells

**Figure 3:**
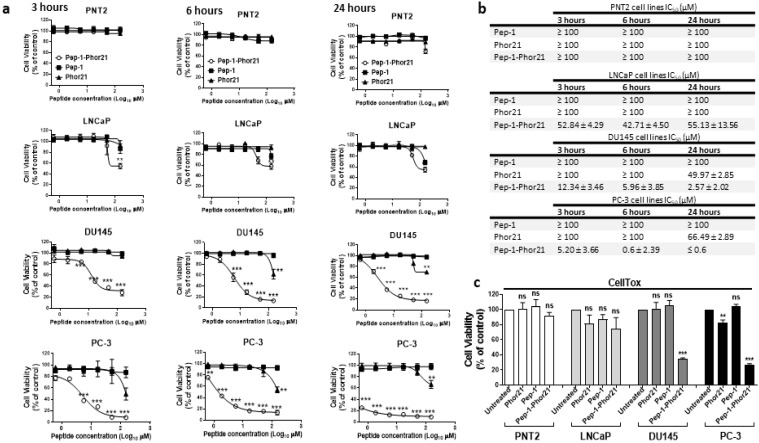
The cytotoxic effect of Pep-1-Phor21 peptide on prostate cancer cells. (**a**) Dose-dependent effect of Pep-1-Phor21 (○), Pep-1 (■) and Phor21 (▲) on the viability of non-IL-13Rα2-expressing cell lines (PNT2 and LNCaP) and IL-13Rα2-expressing cell lines (DU145 and PC-3). The viability of cells treated with 0–150 µM of either Pep-1-Phor21, Phor21 or Pep-1 peptide for 3 h, 6 h or 24 h was assessed using alamarBlue assay. (**b**) The IC_50_ of peptides for various cell lines for different incubation times. (**c**) The effect of Pep-1-Phor21, Pep-1 and Phor21 on the viability of non-IL-13Rα2-expressing and IL-13Rα2-expressing cell lines was assessed using CellTox assay. PNT2 (120 µM), LNCaP (120 µM), DU145 (24 µM) and PC-3 (10 µM) cells were incubated with Pep-1-Phor21, Phor21 or Pep-1 peptide (the concentration used for each cell line is shown next to it in the brackets) for 3 h before their viability was assessed using CellTox assay. The data represent means ± SEM (error bars represent SEM) of three independent experiments (** *p* < 0.01; *** *p* < 0.001; ns, not significant).

Since IL-13Rα2 expression is relatively high in metastatic prostate cancer cells, we assessed in vitro the potential of Pep-1-Phor21 as a therapeutic drug for prostate cancer. Different prostate non-cancerous (PNT2) and cancerous cell lines (LNCaP, DU145 and PC-3) were grown as monolayers (2D culture), treated with 0–150 µM of Pep-1 (ligand), Phor21 or Pep-1-Phor21 for 3 h, 6 h or 24 h and assessed for cell viability via the alamarBlue assay (Figure 3a). The treatment of PNT2 cells, which express very low levels of IL-13Rα2, with Pep-1-Phor21 for up to 24 h did not affect the cell viability. However, LNCaP cells, which also express very low levels of IL-13Rα2, showed a reduction in cell viability only with very high concentrations of the peptide and the IC_50_ was calculated as ~50 µM (Figure 3b). DU145 and PC-3 cells, which express relatively higher levels of IL-13Rα2, were more sensitive to Pep-1-Phor21 (Figure 3a), with IC_50_ values reduced as the incubation time increased (Figure 3b). The unconjugated peptides Phor21 and Pep-1 had little effect on the viability of prostate cancer cells except that PC-3 and DU145 cells showed a reduction in viability in the presence of a high concentration of Phor21. Further, when we down-regulated IL-13R α2 expression in the cancer cells via siRNA treatment, they lost sensitivity to Pep-1-Phor21, confirming that this hybrid peptide kills the cancer cells by targeting IL-13Rα2 (Figure S1). Since the 3 h treatment seems to be optimal for analysing the dose-dependent effect of the peptide on the tumour cell lines used in the study, we used the treatment of peptides for 3 h in further experiments unless otherwise indicated. The cytotoxic effect of the peptides on prostate cancer cell lines was also assessed by incubating cells with the peptides for 3 h (Figure 3c). As seen with the cell viability assay, Pep-1-Phor21 was more cytotoxic towards DU145 and PC-3 than Phor21 alone. Again, Pep-1-Phor21 had little effect on PNT2 and LNCaP cells. Together, these results suggest that Pep-1-Phor21 can rapidly and selectively kill cells expressing IL-13Rα2.

### 3.4. Characterisation of the Mode of Action of Pep-1-Phor21

**Figure 4:**
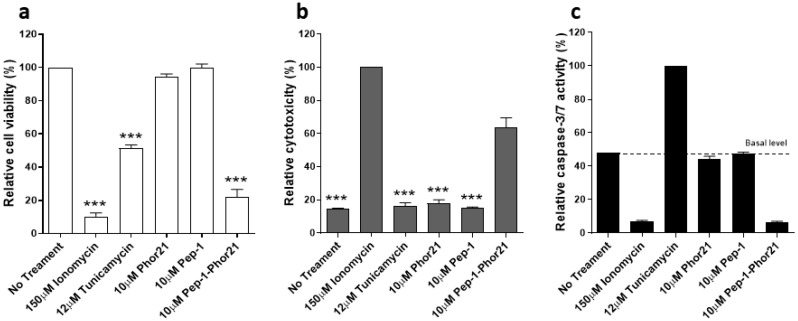
Characterisation of Pep-1-Phor21 mode of action. PC-3 cells were treated with Pep-1, Phor21, Tunicamycin (positive control for apoptosis), Ionomycin (positive control for necrosis (cytotoxicity)) or Pep-1-Phor21 for 6 h and cell viability (**a**), necrosis (**b**) and apoptosis (**c**) were assessed. Untreated (**a**), Ionomycin-treated (**b**) and Tunicamycin-treated cells (**c**) were considered to be 100%. The data represent means ± SEM (error bars represent SEM) of three independent experiments with three different passages of the respective cell line (*** *p* < 0.001).

To understand how Pep-1-Phor21 affects the viability of cancer cells in vitro, PC-3 cells were incubated with Pep-1, Phor21 or Pep-1-Phor21 for 6 h and cell viability, apoptosis and necrosis were assessed (Figure 4). Tunicamycin and ionomycin were used as positive controls for cell apoptosis and cell necrosis, respectively. As expected, unconjugated peptides (Pep-1 and Phor21) did not affect PC-3 cells in all three assays, whereas Pep-1-Phor21 significantly reduced cell viability (Figure 4a) and caused cell necrosis (Figure 4b) but not cell apoptosis (Figure 4c), indicating that Pep-1-phor21 reduces PC-3 cells’ viability through cell necrosis.

### 3.5. Analysis of IL-13Rα2 Expression in Prostate Cancer Cells Treated with TSA or 5-aza-dC

**Figure 5:**
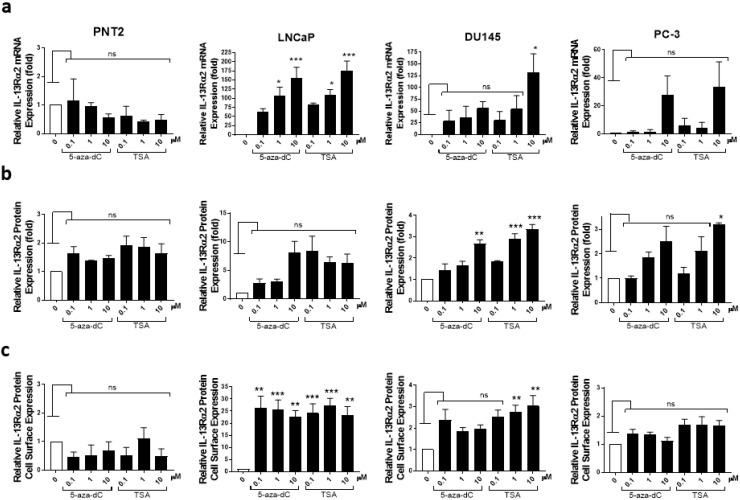
Analysis of IL-13Rα2 expression in prostate cancer cell lines treated with TSA or 5-aza-dC. A non-cancer cell line (PNT2) and cancer cell lines (LNCaP, DU145 and PC-3) were treated with 0.1, 1 and 10 µM of TSA or 5-aza-dC for 24 h. (**a**) RT-PCR analysis of IL-13Rα2 mRNA expression in non-cancer and cancer cell lines. (**b**) Western blot analysis of IL-13Rα2 protein expression in non-cancer and cancer cell lines. Quantification of IL-13Rα2 protein expression was performed via densitometric analysis and normalising to housekeeping protein (α-Tubulin) expression. (**c**) Cell surface expression of anti-IL-13Rα2 was assessed by subjecting non-permeabilised cells to ELISA. All Western blots and RT-PCR values are normalised to housekeeping controls. The data are mean ± SEM (error bars represent SEM) values of three independent experiments. IL-13Rα2 expression levels in cells treated with TSA or 5-aza-dC were compared with those in non-treated control cells (* *p* < 0.05; ** *p* < 0.01; *** *p* < 0.001; ns, not significant).

IL-13Rα2 gene expression is epigenetically regulated in pancreatic cancer (Fujisawa et al., 2011). Therefore, we analysed whether epigenetic modification agents, TSA (a histone deacetylase inhibitor) and 5-aza-dC (a DNA methyltransferase inhibitor) had any effect on IL-13Rα2 expression in prostate cell lines (Figure 5). Prostate non-cancerous (PNT2) and cancerous cell lines (LNCaP, DU145 and PC-3) were treated with 0–10 µM TSA or 5-aza-dC for 24 h and IL-13Rα2 mRNA levels were analysed via RT-PCR; total protein levels were analysed via immunoblotting, whereas cell surface protein expression was measured via ELISA. There was no considerable change in IL-13Rα2 mRNA or protein expression in PNT2 cells (a non-cancerous cell line with undetectable levels of IL13Rα2) after treatment with TSA or 5-aza-dC. However, LNCaP cancer cells, which normally express very low levels of IL-13Rα2, showed increased expression of IL-13Rα2 mRNA (Figure 5a) and protein (Figure 5b) and increased cell surface expression of IL-13Rα2 (Figure 5c) following treatment with TSA or 5-aza-dC. The expression of IL-13Rα2 mRNA and protein was also further increased in DU145 and PC-3 cell lines after TSA or 5-aza-dC treatment, indicating that IL-13Rα2 expression is epigenetically regulated in prostate cancer cell lines.

### 3.6. The Cytotoxic Effect of Pep-1-Phor21 on Prostate Cancer Cells Treated with TSA or 5-aza-dC

**Figure 6:**
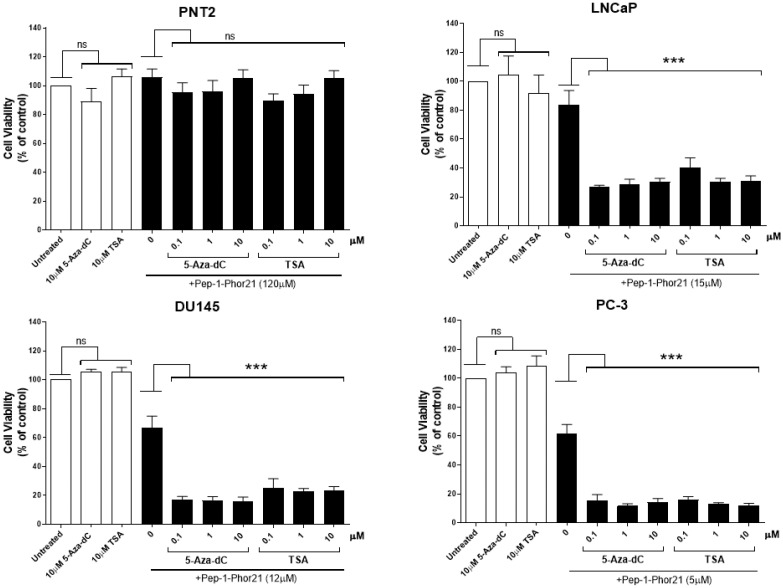
Effect of Pep-1-Phor21 on prostate cell lines treated with TSA or 5-aza-dC. Pep-1-Phor21 sensitivity of no- or low-IL-13Rα2-expressing (PNT2 and LNCaP) and high-IL-13Rα2-expressing (DU145 and PC-3) cell lines treated with 0.1, 1 and 10 µM of TSA or 5-aza-dC for 24 h was analysed using the CellTox assay. PNT2 (120 µM), LNCaP (15 µM), DU145 (12 µM) and PC-3 (5 µM) cells were incubated with the Pep-1-Phor21 lytic peptide (the concentration used for each cell line is shown next to it in the brackets) for 3 h before cell viability was assessed using the CellTox assay. The data represent means ± SEM (error bars represent SEM) of data obtained from three independent experiments. Cell viability of cells treated with TSA or 5-aza-dC was compared with that of non-treated control cells (ns, not significant; *p* > 0.05; *** *p* < 0.001).

As TSA or 5-aza-dC treatments increase IL-13Rα2 expression in prostate cancer cells, we determined whether treatment with these epigenetic modulators also altered the sensitivity of prostate cancer cells to Pep-1-Phor21. The cytotoxicity of Pep-1-Phor21 towards cell lines that express low levels of IL-13Rα2 (PNT2 and LNCaP) and cell lines that express higher levels of IL-13Rα2 (DU145 and PC-3), which had been treated with TSA and 5-aza-dC, was assessed in vitro using the CellTox assay (Figure 6). Cells were treated with 0.1, 1 and 10 µM of TSA or 5-aza-dC for 24 h prior to 3 h of treatment with Pep-1-Phor21 (Figure 6). The concentration of peptide used for each cell line (120 µM for PNT2 cells, 15 µM for LNCaP, 12 µM for DU145 and 5 µM for PC-3) was based on its IC_50_ in the alamarBlue assay (Figure 3b). TSA and 5-aza-dC did not increase the sensitivity of the non-cancerous cell line (PNT2) to Pep-1-Phor21. However, TSA and 5-aza-dC significantly increased the sensitivity of the low-IL-13Rα2-expressing cancer cell line (LNCaP) to Pep-1-Phor21. DU145 and PC-3 cell lines, which express high levels of IL-13Rα2, also showed increased sensitivity to Pep-1-Phor21 after treatment with TSA or 5-aza-dC. The results demonstrate that TSA and 5-aza-dC increase the sensitivity of prostate cancer cells to Pep-1-Phor21.

### 3.7. IL-13Rα2 mRNA Expression in 3D-Cultured Prostate Cancer Cells

**Figure 7:**
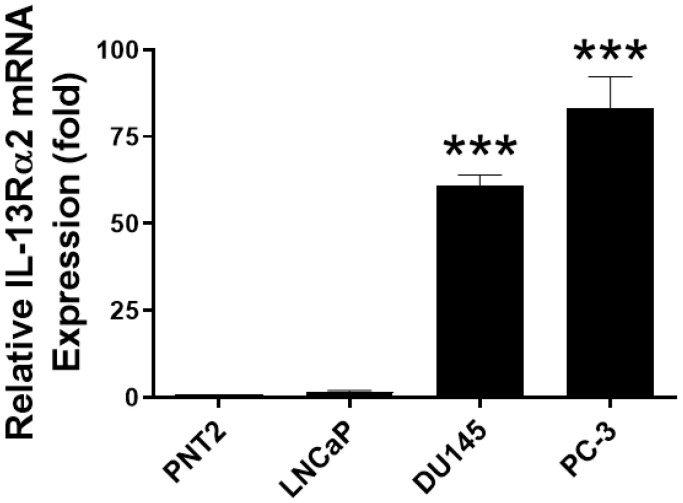
Expression of IL-13Rα2 mRNA in prostate cell lines in 3-dimensional culture. mRNA was isolated from the cells grown in a Terasaki plate to generate a spheroid and RT-PCR was used to quantify mRNA expression using IL-13Rα2 specific primers. The data represent means ± SEM (error bars represent SEM) of three independent experiments. All RT-PCR results were normalised to housekeeping controls. IL-13Rα2 levels in cancerous LNCaP, DU145 and PC-3 cells were compared to those in non-cancerous PNT2 cells (*** *p* < 0.001).

Cancer cells grown in vitro in a 3D environment can resemble the microenvironment of solid tumours and therefore can reveal a more realistic drug response [61]. Thus, we first analysed IL-13Rα2 mRNA expression in prostate non-cancerous (PNT2) and cancerous cell lines (LNCaP, DU145 and PC-3) cultured as 3D spheroids. IL-13Rα2 mRNA expression was observed in prostate cancer cells but was undetectable in PNT2 cells (Figure 7). When compared to IL-13Rα2 mRNA expression in LNCaP cells, DU145 and PC-3 cells expressed high levels of IL-13Rα2 mRNA. Together, these results suggest that the expression pattern of IL-13Rα2 in 3D-cultured prostate cancer cell lines is similar to that observed in 2D-cultured cells.

### 3.8. Effect of Pep-1-Phor21 on the Viability of 3D-Cultured Prostate Cancer Cells

**Figure 8:**
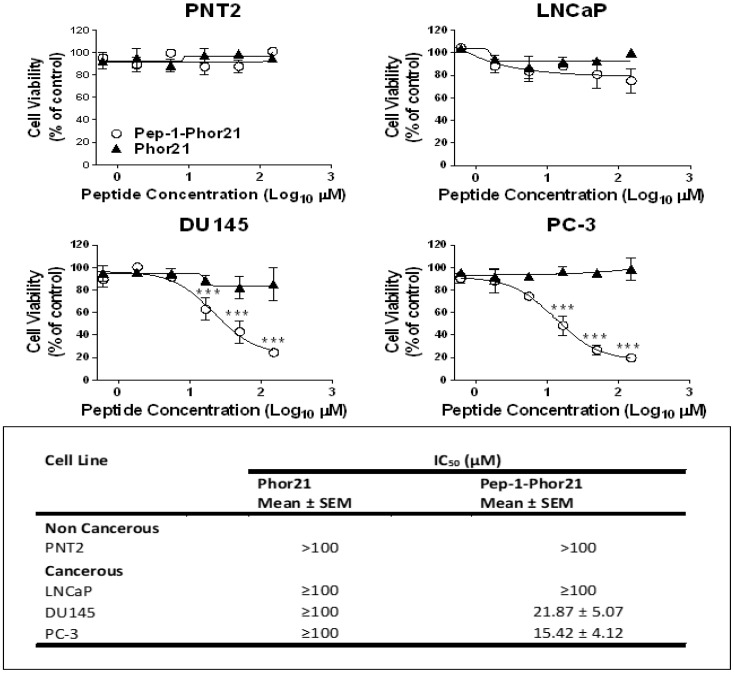
Dose-dependent effect of Pep-1-Phor21 on the viability of 3D-cultured prostate cancer cells assessed using CellTox green assay. Dose-dependent effect of Pep-1-Phor21 and Phor21 on the viability of non-IL-13Rα2-expressing cell lines (PNT2 and LNCaP) and IL-13Rα2-expressing cell lines (DU145 and PC-3). The cells were treated with 0–150 µM of Pep-1-Phor21 or Phor21 peptides for 3 h and the viability of cells was assessed using the CellTox assay. The data represent means ± SEM (error bars represent SEM) of three independent experiments (*** *p* < 0.001).

**Figure 9:**
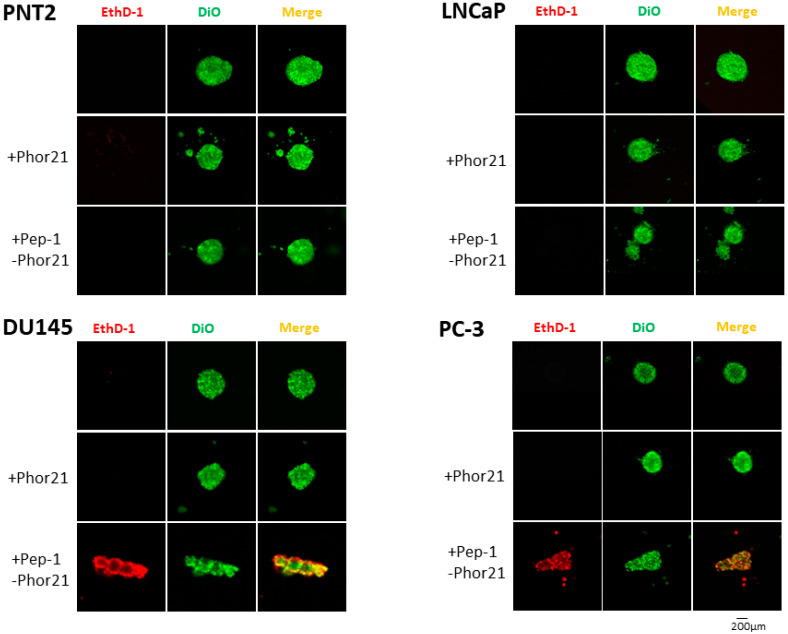
Effect of Pep-1-Phor21 on 3D-cultured prostate cancer cells assessed via live/dead staining. The prostate cancer cells were treated without or with 30 µM of Phor21 or Pep-1-Phor21 for 3 h and the viability of cells was assessed via staining for live (fluorescent vital membrane dye Vybrant DiO, green) and dead (ethidium homodimer I (Ethd-1), red) cells.

The cytotoxic effect of Pep-1-Phor21 peptide on 3D-cultured non-cancerous (PNT2) and cancerous (LNCaP, DU145 and PC-3) prostate cells was determined. For this purpose, cells grown in spheroids were incubated with 0–150 µM Phor21 or Pep-1-Phor21 for 3 h and cell viability was assessed using the CellTox green assay (Figure 8). Pep-1-Phor21 affected the viability of DU145 and PC-3 cells (which express relatively high levels of IL-13Rα2) that had been cultured in spheroids, in a dose-dependent manner (Figure 8). The IC_50_ of Pep-1-Phor21 for these cell lines was <25 µM. In contrast, low-IL-13Rα2-expressing PNT2 and LNCaP cells cultured as 3D spheroids showed little to no loss in cell viability in the presence of Pep-1-Phor21 and the IC_50_ of Pep-1-Phor21 for these cell lines was >100 µM (Figure 8). The unconjugated lytic peptide Phor21 showed little or no effect on the viability of 3D-cultured prostate cancer cell lines used in this study. For qualitative analysis, cell viability was visualised, using confocal fluorescence microscopy, after staining the cells grown in spheroids via live/dead staining (Figure 9). Cells expressing IL-13Rα2 (DU145 and PC3) treated with Pep-1-Phor21 exhibited stronger EthD-1 staining, indicative of cell death, and more so than the Phor21-treated and untreated cells. These results provide both qualitative and quantitative evidence to suggest that 3D-cultured prostate cancer cells with high levels of IL-13Rα2 are more sensitive to Pep-1-Phor21.

### 3.9. IL-13Rα2 mRNA Expression in 3D-Cultured Prostate Cell Lines Treated with TSA and 5-aza-dC

**Figure 10:**
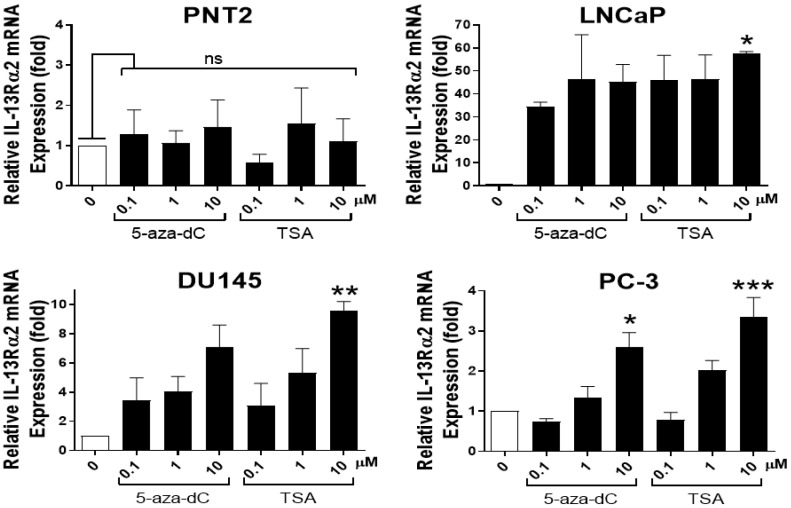
Analysis of IL-13Rα2 mRNA expression in 3D prostate cell lines treated with TSA and 5-aza-dC via RT-PCR. RT-PCR analysis of IL-13Rα2 expression in various prostate cell lines treated with 0.1, 1 and 10 µM of TSA and 5-aza-dC for 24 h. The data represent mean ± SEM (error bars represent SEM) of three independent experiments. All RT-PCR values were normalised to housekeeping controls. IL-13Rα2 mRNA levels in cancer cells were compared to those in the PNT2 non-cancer cell line control (* *p* < 0.05; ** *p* < 0.01; *** *p* < 0.001; ns, not significant).

Since TSA and 5-aza-dC treatment were shown to alter IL-13Rα2 expression in prostate cancer cells grown in 2D culture, we investigated whether the treatments could also alter the expression of IL-13Rα2 in prostate cells grown in 3D culture as cell spheroids. For this, the expression of IL-13Rα2 mRNA was assessed in prostate cell spheroids treated with 0–10 µM TSA or 5-aza-dC for 24 h (Figure 10). LNCaP cancer cell spheroids, which normally have low levels of IL-13Rα2, showed increased expression levels of IL-13Rα2 mRNA, which was significant, with 10 µM of TSA treatment. However, the increase was not as much as seen in TSA- or 5-aza-dC-treated cells grown in a 2D monolayer (Figure 5). A substantial increase in the expression of IL-13Rα2 was also detected in the more aggressive cancer cell lines (DU145 and PC-3) when they were grown as spheroids and treated with TSA or 5-aza-dC.

### 3.10. The Cytotoxic Effect of Pep-1-Phor21 on Prostate Cell Spheroids Treated with TSA and 5-aza-dC

**Figure 11:**
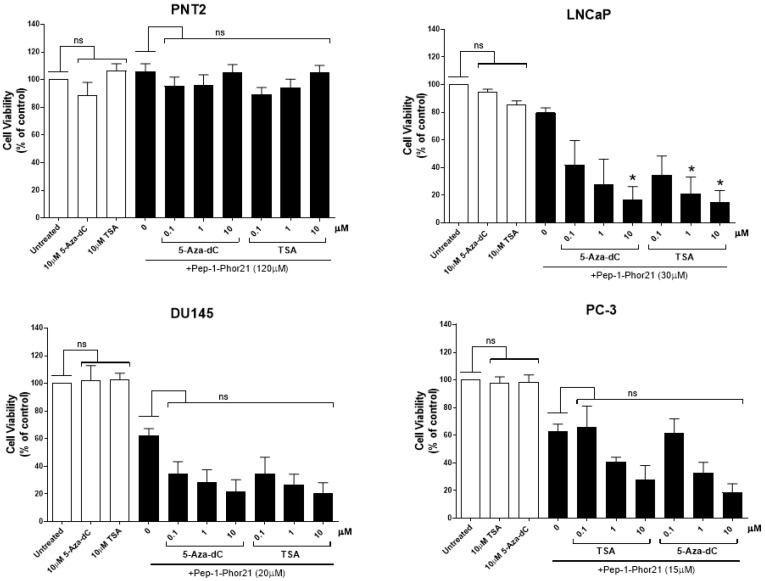
Effect of Pep-1-Phor21 on cell viability of 3D-cultured prostate cell lines treated with TSA or 5-aza-dC. Pep-1-Phor21 sensitivity of no or low-IL-13Rα2-expressing (PNT2 and LNCaP) and high-IL-13Rα2-expressing (DU145 and PC-3) cell lines treated with 0.1, 1 and 10 µM of TSA or 5-aza-dC for 24 h was analysed using the CellTox assay. PNT2 (120 µM), LNCaP (30 µM), DU145 (20 µM) and PC-3 (15 µM) were incubated with Pep-1-Phor21 (the concentration used for each cell line is shown next to it in the brackets) for 3 h before cell viability was assessed using the CellTox assay. The data represent means ± SEM (error bars represent SEM) of data obtained from three independent experiments. The cell viability of treated cells compared to that of non-treated control cells (* *p* < 0.05; ns, not significant).

As TSA and 5-aza-dC treatments increased IL-13Rα2 expression in prostate cancer cells grown in 3D culture, the effect of TSA or 5-aza-dC treatment on the sensitivity of prostate cancer cell spheroids to Pep-1-Phor21 was determined in vitro using the CellTox assay. For this purpose, cell lines expressing no or little IL-13Rα2 (PNT2 and LNCaP) and those expressing high levels of IL-13Rα2 (DU145 and PC-3) were grown as cell spheroids and treated with 0.1, 1 and 10 µM TSA or 5-aza-dC for 24 h prior to incubation for 3 h with Pep-1-Phor21. The concentration of peptide used for each cell line (120 µM for PNT2 cells, 30 µM for LNCaP, 20 µM for DU145 and 15 µM for PC-3) was based on the IC_50_ for inhibition of viability of 3D-cultured cells given in Figure 8. The treatment with TSA or 5-aza-dC did not increase the sensitivity of the non-cancerous cell line PNT2 to Pep-1-Phor21 (Figure 11). However, TSA and 5-aza-dC treatment considerably increased the sensitivity of the low-IL-13Rα2-expressing cancerous cell line (LNCaP) to Pep-1-Phor21. DU145 and PC-3 cell lines (which express high levels of IL-13Rα2) that had been grown as spheroids also showed increased sensitivity to Pep-1-Phor21 when they had been treated with TSA or 5-aza-dC. The results demonstrated that TSA and 5-aza-dC increase the sensitivity of prostate cancer cells grown in 3D spheroids to Pep-1-Phor21.

## 4. Discussion

In this study, we have described a new peptide drug that specifically targets IL-13Rα2 on the surface of prostate cancer cell lines and thereby becomes toxic to these cells. Since its discovery as an overexpressed protein in glioblastoma multiforme (GBM), IL-13Rα2 has become an attractive therapeutic target for cancer [27]. As a result, many IL-13-conjugated cytotoxic agents targeting the receptor have been developed [27]. However, these agents also recognize the widely expressed IL-13Rα1, indicating that there is an unmet need for specific IL-13Rα2 targeting agents [38,42,62,63]. To improve the specificity of IL-13Rα2 targeting, a peptide (Pep-1) that exclusively binds to IL-13Rα2 at a different region from that of the native ligand has been used in this study [40]. We have covalently conjugated Pep-1 to the Phor21 lytic peptide and used it as a cytotoxic drug against prostate cancer cells.

In this study, our results have demonstrated altered expression of IL-13Rα2 in prostate cancer tissues and cell lines. The expression of IL-13Rα2 was observed to be significantly higher in the metastatic prostate cancer cell lines DU145 and PC-3 than in either the non-cancerous prostate cell line PNT2 or the non-metastatic prostate cancer cell line LNCaP. Importantly, IL-13Rα2 mRNA expression levels in prostate cell lines correlated with levels in prostate cancer tissue samples and healthy tissue. Our results agree with previous studies, which demonstrated that the expression of IL-13Rα2 is high in tumorigenic and metastatic prostate cancer cells [12,13,64]. Altogether, the data indicate that IL-13Rα2 is expressed in prostate cancer and could be used as a possible therapeutic target. In this study, we have demonstrated that Pep-1-Phor21 has therapeutic applicability by targeting cancer cells expressing IL-13Rα2. The non-cancerous cell line (PNT2) exhibited low or undetectable levels of IL-13Rα2 expression, and correspondingly showed little or no sensitivity to the Pep-1-Phor21 lytic peptide conjugate, whereas cancerous cell lines (DU145 and PC-3), which express IL-13Rα2 at very high levels, showed high sensitivity to the Pep-1-Phor21 peptide. In addition, only a 3 h exposure to Pep-1-Phor21 was needed to reduce cell viability significantly in DU145 and PC-3 cells.

The decision to assess the efficacy of epigenetic modulators in combination with Pep-1-Phor21 was based on the fact that the epigenetic modulators have been shown by two separate studies to upregulate IL-13Rα2 expression [38,60]. We also showed, in this study, that histone deacetylation and DNA methylation inhibitors upregulated IL-13Rα2 in prostate cancer cells, and went further to determine whether this would enhance the sensitivity of the cells to Pep-1-Phor21. Both TSA and 5-aza-dC increased not only IL-13Rα2 expression but also the sensitivity of the cells to Pep-1-Phor21. Notably, the effects were seen with LNCaP cells, which normally express low levels of IL-13Rα2 and are sensitive only to high concentrations of Pep-1-Phor21, as well as with the metastatic prostate cancer cell lines DU145 and PC-3, which express higher IL-13Rα2 levels and are sensitive to Pep-1-Phor21, even in the absence of TSA or 5-aza-dC treatment. TSA and 5-aza-dC did not affect the IL-13Rα2 expression levels or sensitivity towards Pep-1-Phor21 of the non-cancerous prostate cell line PNT2, which does not express any detectable levels of IL-13Rα2. Taken together, these results suggest that a combination treatment consisting of the epigenetic modulator(s) and Pep-1-Phor21 would be able to target heterogeneous prostate cancers (increasing the levels of IL-13 Rα2 of low-expressing cells and then targeting them with Pep-1-Phor21 whilst Pep-1-Phor21 could target the high-expressing cells directly) without damaging the surrounding normal tissues.

One possible reason for the lack of effect of TSA/5-aza-dC and Pep-1-Phor21 on the non-cancerous prostate cell line PNT2 is that TSA and 5-aza-dC induction of IL-13Rα2 requires the AP-1/c-JUN pathway which might be inactivated in normal cells [38]. A possible link has been suggested between c-JUN and c-FOS expression and IL-13Rα2, which is consistent with the expression profile of human IL-13 Rα2 in malignant gliomas [37,65]. c-JUN and c-FOS, both well-established oncogenes, are considered to play a critical role in tumorigenesis, proliferation and transformation, angiogenesis, tumour invasion and metastasis, and their expression is associated with poor clinical outcomes [66]. It has been shown that PC-3 cells express higher levels of c-JUN and c-FOS compared to LNCaP cells, which in turn express higher levels than normal prostate cell lines [67,68]. This could explain why IL-13Rα2 expression is lower in LNCaP cells compared to PC-3 and also why the addition of TSA and 5-aza-dC activates IL-13Rα2 expression in these cells.

To closely mimic the pathophysiology of in vivo tumours, we investigated the efficacy of Pep-1, Phor21 and Pep-1-Phor21 on PNT2, LNCaP, DU145 and PC-3 cells grown as 3D spheroids. Only Pep-1-Phor21 showed any anticancer activity in the spheroids of IL-13Rα2-expressing cells. These results correlated with those obtained from cells grown in the 2D cell monolayer model. Actual tumours contain extracellular matrix (ECM), which can reduce the efficiency of drugs due to the molecules’ inability to penetrate through the complex matrix and reach the targeted cancer cells; however, Pep-1-Phor21 showed no such limitation, confirming that these peptides can penetrate with or without the presence of ECM. Noticeably, the efficiency of the peptides was lower in cell spheroids compared to cells grown as 2D cultures. This could be because IL-13Rα2 expression was demonstrated in this study to be slightly reduced in prostate cells grown as multicellular spheroids when compared to those grown in 2D monolayers. Since the central layers of multicellular spheroids are hypoxic [69,70], the reduction in IL-13Rα2 expression in prostate cancer spheroids could be due to the lack of oxygen. This is supported by a previous report, which suggested that IL-13Rα2 mRNA expression in glioblastoma cells is reduced under hypoxic conditions [71].

One of the biggest issues regarding drug efficiency is the microenvironment of the tumour cells. Cells that are in ECM have been reported to become drug-resistant, enhancing tumour survival [72,73,74]. In one such case, the prostate cancer cell line (PC-3) was shown to be resistant to doxorubicin and paclitaxel, preventing drug-induced apoptosis [72]. The upregulation of ATP-binding cassette transporters, such as P-glycoprotein, and the multidrug-resistance-associated protein is a well-documented mechanism that causes resistance. However, a unique feature of lytic peptides is the total lysis of the cancer cells. The lytic peptide interacts with and permeates the plasma membrane after a threshold concentration of peptides has been reached, thus preventing any drug resistance [46,75].

## 5. Conclusions

We have employed a lytic peptide Pep-1-Phor21 to target prostate cancer cell lines expressing IL-13Rα2. Combining lytic peptide treatment with epigenetic modulators induced cell death more effectively when compared to the efficacies of the treatments alone. More importantly, assays with cells grown as spheroids in vitro to mimic actual tumours showed that Pep-1-Phor21 was able to traffic into the spheroids and kill cells. Altogether, our results provide evidence that this combinatorial treatment could be used in clinics for treating prostate cancer. It is currently unknown how effective this lytic peptide will be against other cancers overexpressing IL-13Rα2. However, a recent study identified a novel IL-13Rα2-targeted hybrid lytic peptide for effective therapy for glioblastoma, which has also been shown to overexpress IL-13Rα2 [51]. In the first study with Pep-1, it was used to target glioblastoma cells. Taken together, these suggest that Pep-1-Phor21 may also be effective against glioblastomas. Studies have shown that IL-13Rα2 is overexpressed in prostate cancer cells, suggesting that it could be used both as a potential biomarker and also as a potentially important therapeutic target for preventing cancer progression [13,76,77].

## Data Availability

Not applicable.

## Supplementary Materials

The following supporting information can be downloaded at https://www.mdpi.com/article/10.3390/biom13020356/s1: Figure S1: Analysis of Pep-1-Phor21 sensitivity of cancer cells treated with IL-13Rα2 siRNA.
