# Supplementary figures and images for: Targeting of the Interleukin-13 Receptor (IL-13R)α2 Expressing Prostate Cancer by a Novel Hybrid Lytic Peptide
Source: Biomolecules. 2023 Feb 12;13(2):356. doi: 10.3390/biom13020356 (PMC9953383; doi:10.3390/biom13020356)

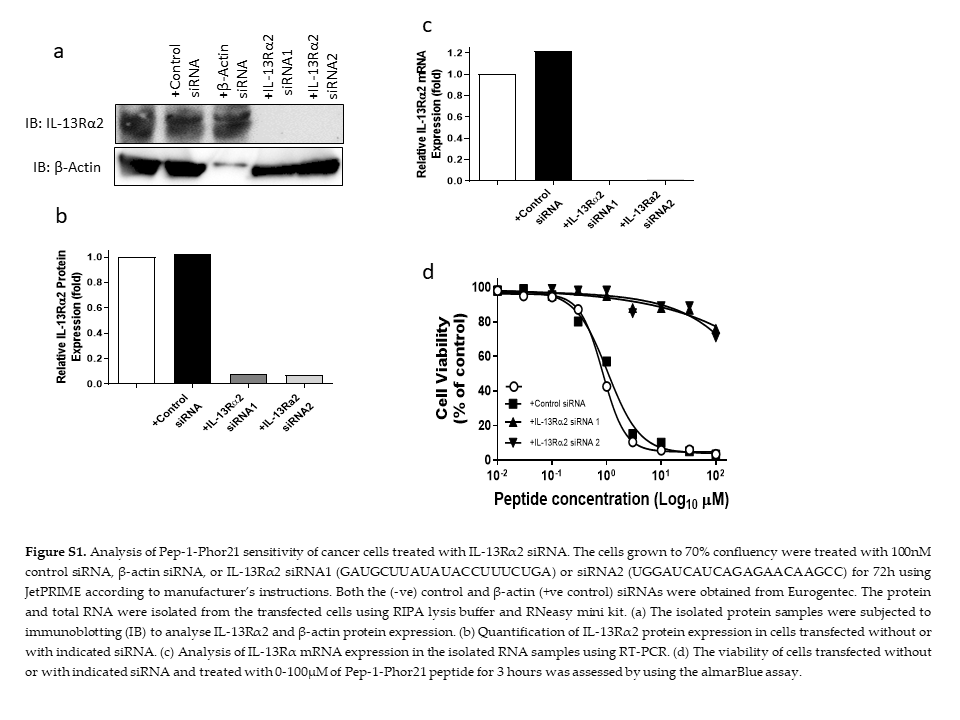

Supplement: Supplementary file 1 [file biomolecules-13-00356-s001.zip › biomolecules-2142293-supplementary.tif]
